# A Narrow Medial Femoral Condyle Width Is Associated With Anteromedial Rotatory Instability After Anterior Cruciate Ligament Injury

**DOI:** 10.1002/ars2.70073

**Published:** 2026-09-10

**Authors:** Shengkun Wu, Ruoxi Zhang, Yu Liu, Yi Zheng, Zhikuan Li, Jiangtao Dong

**Affiliations:** ^1^ The Third Hospital of Hebei Medical University Shijiazhuang Hebei Province, China

## Abstract

**Purpose:**

To identify bone morphological risk factors and injury patterns of medial structural injuries in patients with anteromedial rotatory instability (AMRI) after anterior cruciate ligament (ACL) injury.

**Methods:**

This comparative study analyzed patients’ data between August 2024 and June 2025. ACL‐deficient patients with combined injury of ligaments other than the medial collateral ligament or knee fracture were excluded. Patients aged 16 to 55 who were diagnosed with ACL rupture on magnetic resonance imaging and Kellgren‐Lawrence grade < II on radiograph were included. All ACL injury patients were divided into 2 groups based on physical examination under anesthesia: 58 patients with a positive anterior drawer and Slocum test under anesthesia served as the AMRI group; 25 patients with a positive anterior drawer test but negative Slocum test under anesthesia served as the isolated ACL injury group (nonanteromedial rotatory instability [NAMRI] group). Preoperative magnetic resonance imaging was used to characterize bone morphological factors, including lateral femoral condyle ratio, medial femoral condyle width (MFCW), notch width, notch width index, coronal tibial slope, medial tibial depth, medial tibial posterior slope, and lateral tibial posterior slope. Medial structural injuries (superficial medial collateral ligament, deep medial collateral ligament [dMCL], and anteromedial retinaculum [AMR]) were assessed via ultrasound and magnetic resonance imaging. All patients did not experience postoperative infections or other surgical complications during at least 3 months of follow‐up. During data analysis, t‐tests were used to compare the differences between the mean values of various factors, 1‐way analysis of variance was used to test the influence of each factor, and receiver operating characteristic (ROC) curves were used to test the predictive performance of MFCW.

**Results:**

The comparative study included 141 patients who were divided into 3 groups: AMRI (n = 58), ACL‐injured patients without AMRI (NAMRI, n = 25), and isolated meniscus tear patients (control, n = 58). The AMRI group exhibited significantly smaller MFCW (AMRI: 6.72 ± 0.55 vs control: 7.35 ± 0.51, *P* < .001) and notch width (AMRI: 1.79 ± 0.29 vs control: 2.01 ± 0.37, *P* = .001), whereas significantly larger lateral femoral condyle ratio (AMRI: 0.672 ± 0.039 vs control: 0.644 ± 0.043, *P* = .001), medial tibial depth (AMRI: 0.36 ± 0.07 vs control: 0.31 ± 0.06, *P* < .001), and lateral tibial posterior slope (AMRI: 8.5 ± 2.6 vs control: 6.4 ± 2.9, *P* < .001) relative to the control group. The NAMRI group also showed significant differences, including larger lateral femoral condyle ratio (NAMRI: 0.668 ± 0.035 vs control: 0.644 ± 0.043, *P* = .048), lateral tibial posterior slope (NAMRI: 8.1 ± 2.3 vs control: 6.4 ± 2.9, *P* = .027), and medial tibial depth (NAMRI: 0.36 ± 0.08 vs control: 0.31 ± 0.06, *P* = .004) and smaller MFCW (NAMRI: 7.03 ± 0.37 vs control: 7.35 ± 0.51, *P* = .018), notch width (NAMRI: 1.73 ± 0.24 vs control: 2.01 ± 0.37, *P* = .001), and notch width index (NAMRI: 0.248 ± 0.035 vs control: 0.273 ± 0.044, *P* = .018) when compared with the control group. Critically, the AMRI group exhibited a smaller MFCW (NAMRI: 7.03 ± 0.37 vs AMRI: 6.72 ± 0.55, *P* = .033) relative to the NAMRI group, which represented a key factor in controlling anteromedial rotatory stability. Ultrasonography revealed a high prevalence of dMCL (89.7%) and AMR (63.8%) injuries in the AMRI group, and the combined injury of dMCL and AMR had the most frequent injury pattern. In addition, the ROC curve showed that MFCW showed a higher area under the ROC curve value in AMRI when compared with the control and NAMRI groups, respectively (area under the ROC curve: 0.791 AMRI vs control; 0.655 AMRI vs NAMRI).

**Conclusions:**

A smaller MFCW is associated with AMRI in patients with dMCL and AMR injuries compared with NAMRI patients. The injury pattern in AMRI is predominantly characterized by combined lesions of the ACL and dMCL + AMR.

**Level of Evidence:**

Level IV, retrospective comparative case series.

## Graphical abstract

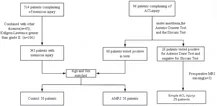

Anterior cruciate ligament (ACL) injuries frequently occur in combination with damage to the medial structures of the knee, leading to a complex condition characterized by anterior translation and rotational instability of the knee.^1^ This pathological condition was defined by Slocum as anteromedial rotatory instability (AMRI).^2^ Evidence indicates that AMRI has more severe clinical implications than isolated ACL injuries, adversely affecting both short‐term return‐to‐sport outcomes and long‐term risks of cartilage degeneration.^3^, ^4^ Consequently, research into AMRI is of paramount importance.

Conventionally, AMRI has been attributed to an ACL injury combined with a grade III injury of the superficial medial collateral ligament (sMCL) and/or the posteromedial complex.^5^ Surgical approaches involving ACL reconstruction alongside posteromedial complex/sMCL repair or reconstruction have been reported to effectively restore knee stability in anterior translation and valgus.^6^, ^7^ Although combined ACL and sMCL reconstruction controlled internal rotation and anterior tibial translation, it failed to restore stability in external rotation.^8^ This suggests the involvement of structures beyond the sMCL in AMRI. In recent years, the anteromedial retinaculum (AMR) has been implicated, with clinical studies reporting concomitant AMR injuries in 72.5% to 73.7% of ACL‐injured patients.^9^ A growing consensus suggests that multiple dynamic and static stabilizers within the medial knee complex contribute to this condition.^1^, ^9^, ^10^, ^11^ Thus, the precise structural determinants of AMRI remain elusive, complicating both its understanding and clinical management.

The clinical diagnosis of AMRI primarily relies on the Slocum test.^10^, ^12^ However, its utility is limited in acute, nonanesthetized patients because of suboptimal sensitivity and specificity, hindering the early identification of AMRI. Meanwhile, specific knee bone morphology is an established risk factor for both primary ACL injury and reconstruction failure. These included the lateral femoral condyle ratio (LFCR), medial femoral condyle width (MFCW), notch width (NW), notch width index (NWI), coronal tibial slope (CTS), medial tibial depth (MTD), medial tibial posterior slope (MTS), and lateral tibial posterior slope (LTS).^13^, ^14^, ^15^, ^16^, ^17^, ^18^ Whether these morphological factors are similarly or distinctly associated with AMRI, compared with general ACL injury, is unknown. Among available imaging modalities, magnetic resonance imaging (MRI) and ultrasound offer superior detail for assessing knee morphology and periarticular soft tissues compared with radiography or computed tomography. Therefore, this study used ultrasound, MRI, and physical examination to compare bone morphology among 3 patient groups: those with a positive anterior drawer test and Slocum test (AMRI group), those with a positive anterior drawer test but negative Slocum test (nonanteromedial rotatory instability [NAMRI] group), and a control group with negative findings for both tests (control group). Additionally, injuries to the medial structures were systematically documented in the AMRI group. The purpose of this study is to identify bone morphological risk factors and injury patterns of medial structural injuries in patients with AMRI after ACL injury.

We hypothesized that the AMRI condition is associated with larger LFCR, MTS, and LTS, as well as smaller MFCW and NW.

## METHODS

This comparative study analyzed patients’ data at the Third Hospital of Hebei Medical University between August 2024 and June 2025. Patients were included and excluded according to the following criteria. ACL‐deficient patients with combined injury of ligaments other than the medial collateral ligament (MCL), posterior cruciate ligament (PCL), anterolateral ligament, and lateral collateral ligament, periarticular knee fracture, or ACL avulsion fracture were excluded. Patients aged 16 to 55 who were diagnosed with ACL rupture on MRI and had a Kellgren‐Lawrence grade < II on radiograph were included. A single senior orthopaedic surgeon (J.D.) performed physical examinations (including anterior drawer test and Slocum test) under anesthesia. ACL‐deficient patients underwent ACL reconstruction and were categorized into 2 groups based on examination findings: patients with a positive anterior drawer and Slocum test under anesthesia served as the AMRI group; patients with a positive anterior drawer test but negative Slocum test under anesthesia served as the isolated ACL injury group (NAMRI group). The control patients were selected from individuals with isolated minor meniscal tear and were individually matched to the AMRI patients by age and sex. Patients were screened according to the following inclusion and exclusion criteria (Figure 1).

**FIGURE 1:**
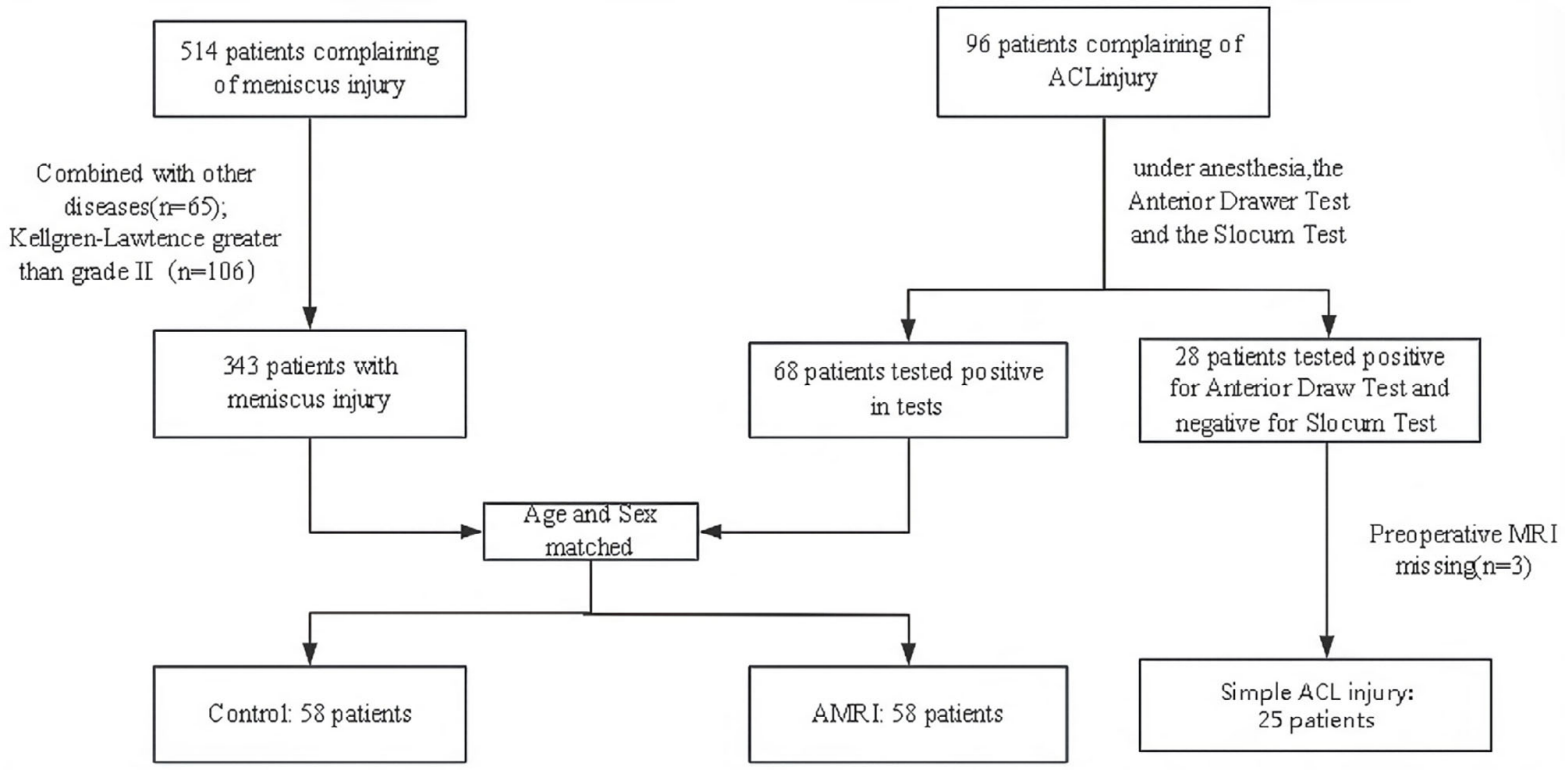
Flowchart of patient enrollment. (ACL, anterior cruciate ligament; AMRI, anteromedial rotatory instability; MRI, magnetic resonance imaging.)

### MRI and Ultrasound Scan and Assessment

All patients underwent 3.0‐T MRI scanning in the supine position with the knee fully or nearly fully extended and toes pointing upward. Scans included axial, sagittal, and coronal planes. During the ultrasound examination, patients were placed in a supine position with the knee flexed at 30° and rotated externally as much as possible.

Patients’ sex, age, and body mass index were recorded. Preoperative MRI was used to measure LFCR, MFCW, NW, NWI, CTS, MTD, MTS, and LTS. Preoperative MRI and ultrasound were performed to assess injured structures: sMCL and deep MCL (dMCL). The damage to the patient's AMR was recorded in the preoperative ultrasound.

MRI data for all included cases were collected and measured using ICPACS software. Meniscal injuries were independently assessed by 2 experienced orthopaedic surgeons (Y.Z., Z.L.) using the 3‐grade T2 signal staging system mentioned by Fischer et al. on MRI: grade I, punctate localized high signal; grade II, linear high signal not extending to the articular surface; and grade III, high signal extending to at least 1 articular surface. Meniscal injuries graded ≥ II were defined as significant meniscal tear.^19^ Only patients with meniscal injuries below grade II were included.

The LFCR was measured according to the method described by Kim et al.^17^ Briefly, a central sagittal MRI slice where the PCL was the most prominent and included the anterior and posterior cortical indentations of the tibia was selected. The anatomical axis of the distal femur was determined by drawing a circle distally tangent to the anterior and posterior femoral cortices and the femoral attachment of the PCL and a circle proximally tangent to the anterior and posterior femoral cortices. The line connecting the centers of these 2 circles defined the femoral anatomical axis (Figure 2A). This axis was then transferred to a T1‐weighted sagittal MRI slice through the lateral femoral condyle. A line connecting the most anterior and posterior points of the lateral femoral condyle was drawn. The LFCR was calculated as the distance from the most posterior point of the femoral condyle to the femoral anatomical axis divided by the total anteroposterior distance of the condyle (Figure 2B).

**FIGURE 2:**
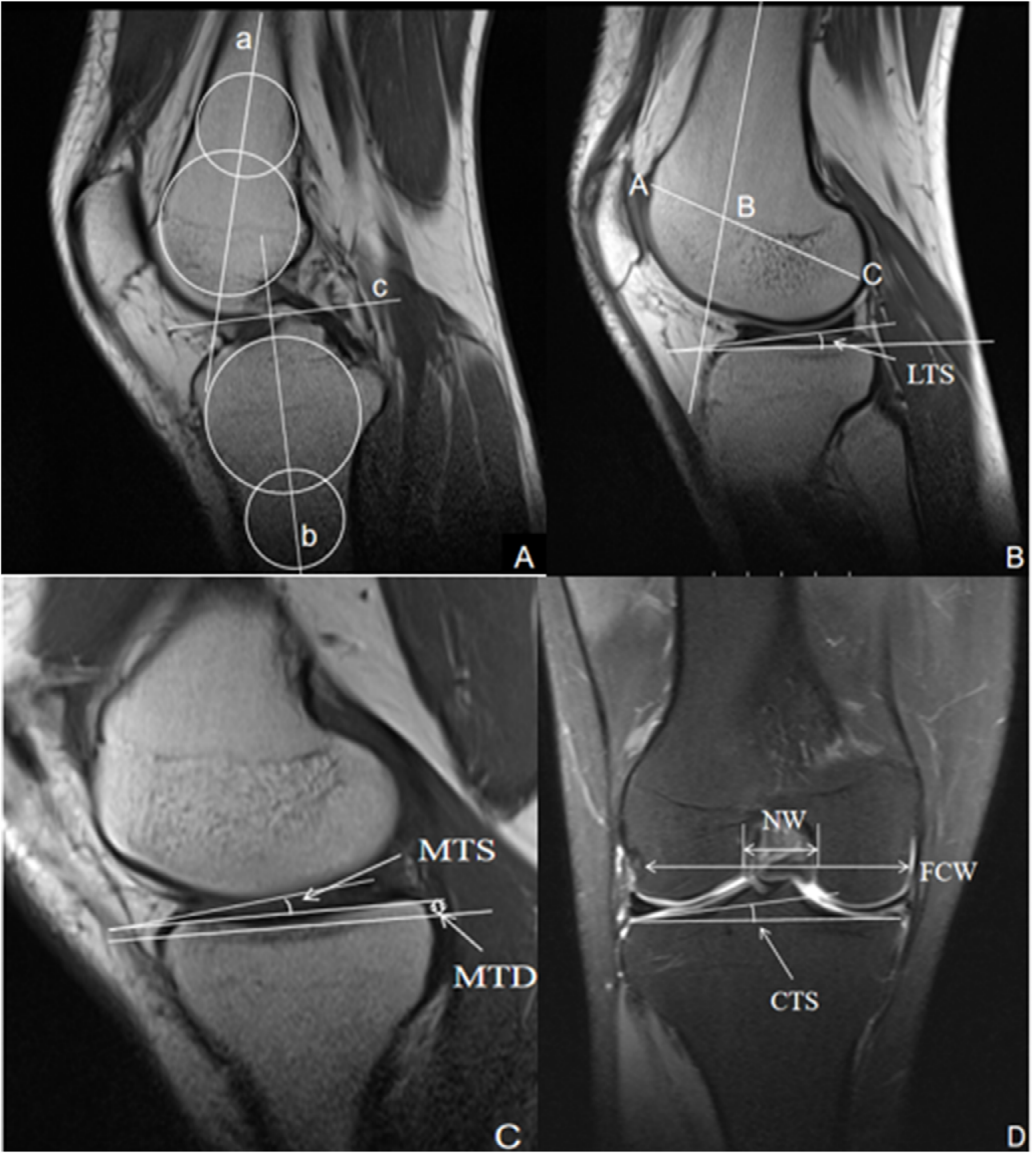
(A) Line “a” is the femoral anatomical axis. Line “b” is the anatomical axis of the tibia. Line “c” is the vertical line of the anatomical axis of the tibia. (B) Line “AC” represents the anterior‐posterior cortical line at the widest part of the lateral condyle of the femur. Point “B” is the intersection of the femoral anatomical axis and line “AC”. BC/AC is the LFCR. LTS was the angle between the tangential plane of the lateral tibial plateau and a line perpendicular to the tibial anatomical axis. (C) MTS was the angle between the tangential plane of the medial tibial plateau and a line perpendicular to the tibial anatomical axis. MTD was the vertical distance between the lowest point of the medial tibial plateau and the tangential plane of the medial tibial plateau. (D) A parallel line of the lowest edge of the femoral condyle. FCW is the width of the femoral condyle at the level of the popliteal groove. NW is the width between the condyles. CTS is the angle between a line connecting the peak points of the medial and lateral tibial plateaus and a line perpendicular to the longitudinal axis of the tibia. (CTS, coronal tibial slope; FCW, femoral condyle width; LFCR, lateral femoral condyle ratio; LTS, lateral tibial posterior slope; MTD, medial tibial depth; MTS, medial tibial posterior slope; NW, notch width.)

NW, MFCW, and NWI were measured according to methods described by Domzalski et al. and Wang et al.^20^, ^21^ Measurements were performed on a coronal MRI slice showing the popliteal groove. A line was drawn connecting the lowest medial and lateral edges of the femoral condyles, and a parallel line was drawn at the level of the popliteal groove. NW was measured as the width between the condyles at this level. MFCW was the distance between the medial and lateral condylar edges (Figure 2D). NWI was calculated as NW/MFCW.

The approach to measure the value of LTS and MTS was referred to a published method.^22^ The tibial anatomical axis was defined by drawing 2 circles on a central sagittal slice of the proximal tibia showing the connection between the PCL and the tibial spine. The proximal circle matched the anterior, posterior, and proximal cortical boundaries, whereas the distal circle matched the anterior and posterior cortices. The line connecting the centers of these circles defined the tibial anatomical axis (Figure 2A). MTS and LTS were measured on sagittal slices through the center of the medial and lateral tibial plateaus, respectively. The tangent line for the lateral plateau was defined along the uppermost cortical edge. The tangent line for the medial plateau was defined by connecting the uppermost anterior and posterior cortical edges (excluding articular cartilage). MTS and LTS were the angles between these respective tangents and a line perpendicular to the tibial anatomical axis (Figure 2B,C). MTD was measured on the same slice used for MTS according to Hudek et al. by constructing a line connecting the peak points of the superior and inferior aspects of the medial tibial plateau.^22^ A parallel line was drawn tangent to the lowest point of the concavity, representing the lowest border of the subchondral bone. The vertical distance between these lines represented the MTD (Figure 2C).

CTS was measured referencing Shultz and Schmitz on the same coronal slice used for measuring NW and MFCW.^23^ CTS was defined as the angle between a line connecting the peak points of the medial and lateral tibial plateaus and a line perpendicular to the longitudinal axis of the tibia (Figure 2D). The slope was considered positive if the medial plateau contact point was lower than the lateral.

Based on the knee anteromedial anatomical region proposed by Peez et al. preoperative ultrasound examination was performed on the anatomical region of the MCL in all AMRI patients.^24^ In the 1 o'clock direction of the MCL femoral insertion point, a distinct ligament‐like structure, termed the AMR, was identified. Injuries to the knee structures—namely, the sMCL, dMCL, and AMR—were recorded (Figure 3).

**FIGURE 3:**
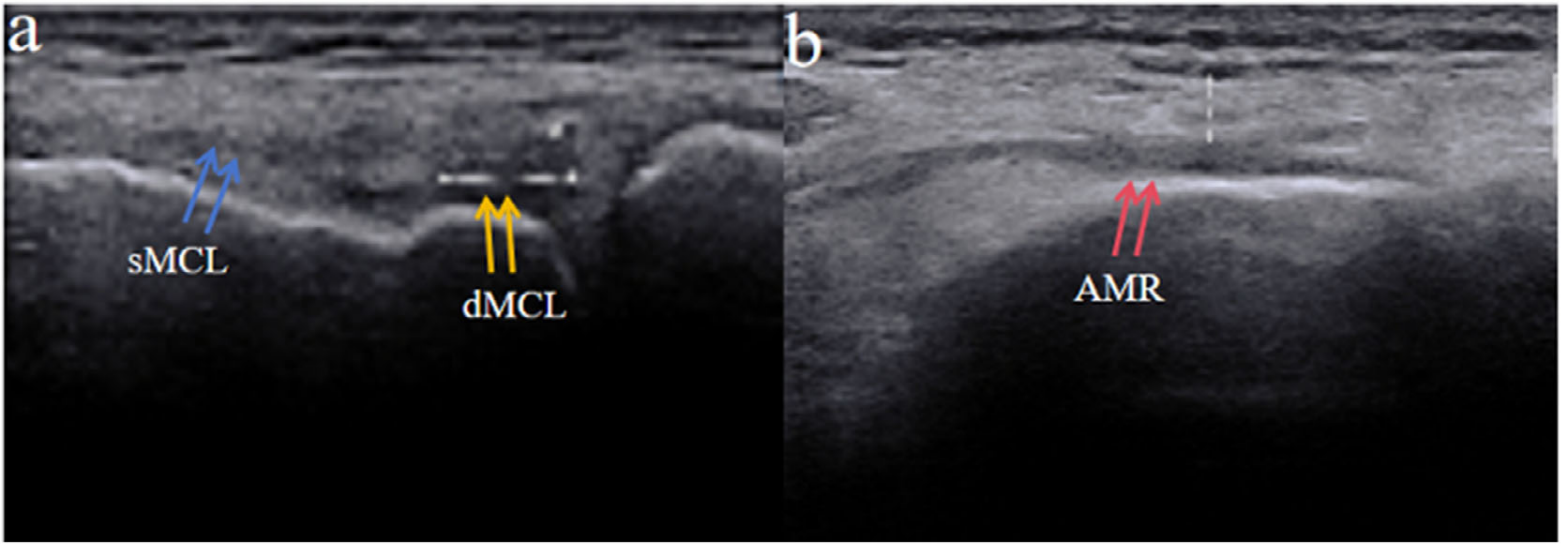
(a) Ultrasound imaging of the medial knee structures shows the sMCL (blue), the dMCL (yellow). (b) Ultrasound imaging of the AMR (red). (AMR, anteromedial retinaculum; dMCL, deep medial collateral ligament; sMCL, superficial medial collateral ligament.)

### Statistical Analysis

IBM SPSS and GraphPad Prism were used for statistical analysis. One‐way analysis of variance was used to analyze differences in bone morphological parameters among the 3 groups. Independent sample t‐tests were used to analyze significant differences between any 2 groups. A *P* value < .05 was considered indicative of a statistically significant difference. Receiver operating characteristic (ROC) curve analysis was used to determine cutoff values for morphological parameters that showed significant differences among groups.

### Ethical Approval

This study was approved by the Ethics Committee of the Third Hospital of Hebei Medical University (2024‐127‐1).

## RESULTS

The intraobserver reliability of measurements is shown in Table 1. The intraclass correlation coefficient  for the LFCR was 0.91 (95% confidence interval [CI], 0.88‐0.96), the CTS was 0.78 (95% CI, 0.70‐0.84), the NW was 0.87 (95% CI, 0.79‐0.95), the MFCW was 0.97 (95% CI, 0.92‐0.98), the NWI was 0.89 (95% CI, 0.81‐0.95), the MTD was 0.94 (95% CI, 0.87‐0.97), the LTS was 0.98 (95% CI, 0.95‐0.99), and the MTS was 0.89 (95% CI, 0.81‐0.93).

**TABLE 1:** Intraobserver Reliability Among All MRI Measurements

	**Intraobserver Reliability (95% CI)**
LFCR	0.91 (0.88‐0.96)
CTS	0.78 (0.70‐0.84)
NW	0.87 (0.79‐0.95)
MFCW	0.97 (0.92‐0.98)
NWI	0.89 (0.81‐0.95)
MTD	0.94 (0.87‐0.97)
LTS	0.98 (0.95‐0.99)
MTS	0.89 (0.81‐0.93)

CI, confidence interval; CTS, coronal tibial slope; LFCR, lateral femoral condyle ratio; LTS, lateral tibial posterior slope; MFCW, medial femoral condyle width; MRI, magnetic resonance imaging; MTD, medial tibial depth; MTS, medial tibial posterior slope; NW, notch width; NWI, notch width index.

A total of 58 AMRI patients and 25 NAMRI patients were included. A group of 58 controls was used. The result of ultrasound examination of 58 AMRI patients showed that 16 patients (27.5%) had sMCL injury, 52 patients (89.7%) had dMCL injury, and 37 patients (63.5%) had AMR injury (Figure 4). Among the injury patterns in this population, 31 patients had sustained concurrent injuries to the dMCL and AMR. Combined dMCL and AMR injuries were the most common combined injury.

**FIGURE 4:**
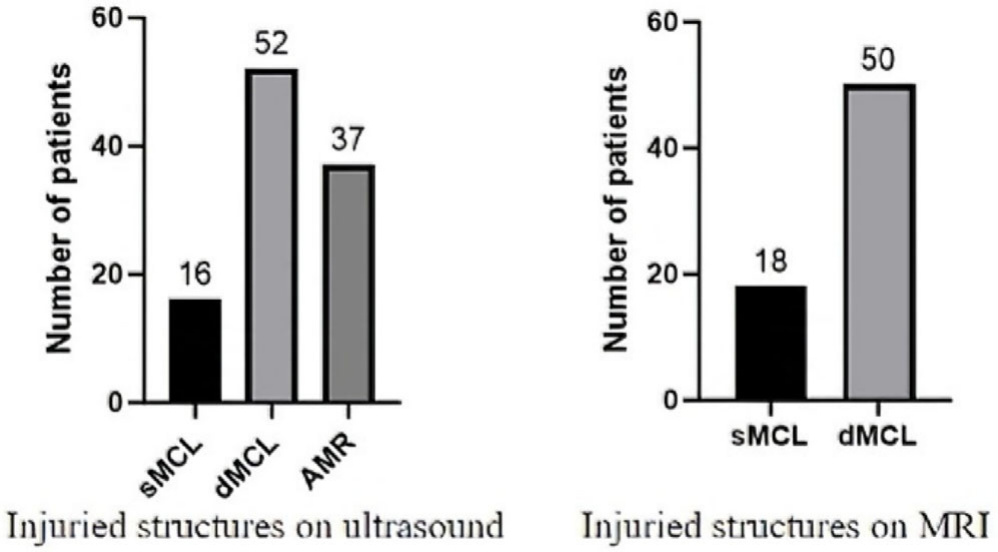
The number of injuries to the sMCL (16), dMCL (52), and AMR (37) was recorded on preoperative ultrasound individually. The number of injuries to the sMCL (18) and dMCL (50) was recorded on preoperative MRI individually. (AMR, anteromedial retinaculum; dMCL, deep medial collateral ligament; MRI, magnetic resonance imaging; sMCL, superficial medial collateral ligament.)

A count of positive findings for sMCL and dMCL injuries between ultrasound and MRI was performed (Table 2). For sMCL injury, MRI identified 18 positive patients (31%), whereas ultrasound identified 16 positive patients (27.5%). For dMCL injury, MRI identified 50 positive patients (86.2%), and ultrasound identified 52 positive patients (89.7%).

**TABLE 2:** Count of Patients With sMCL and dMCL Injuries on Ultrasound and MRI

	**sMCL Injury**	**Non‐sMCL Injury**	**Injury Rate**
MRI	18	40	31.0%
Ultrasound	16	42	27.6%

	**dMCL Injury**	**Non‐dMCL Injury**	**Injury Rate**
MRI	50	8	86.2%
Ultrasound	52	6	89.7%

dMCL, deep medial collateral ligament; MRI, magnetic resonance imaging; sMCL, superficial medial collateral ligament.

The thickness of the AMR structure was measured by ultrasound (Figure 5). The thickness of the AMR was recorded for both the injured and healthy contralateral knees in the AMRI group. Patients were stratified into the acute injury group (<6 weeks) and the chronic injury group (≥6 weeks). This was done to eliminate the influence of acute edema in the injured tissue on the assessment of AMR injury. A comparison between the injured and healthy sides was made within each group. A significant difference in AMR thickness was found between the injured and healthy sides in both the chronic and acute injury groups (all *P* < .05), with the injured side AMR being thicker.

**FIGURE 5:**
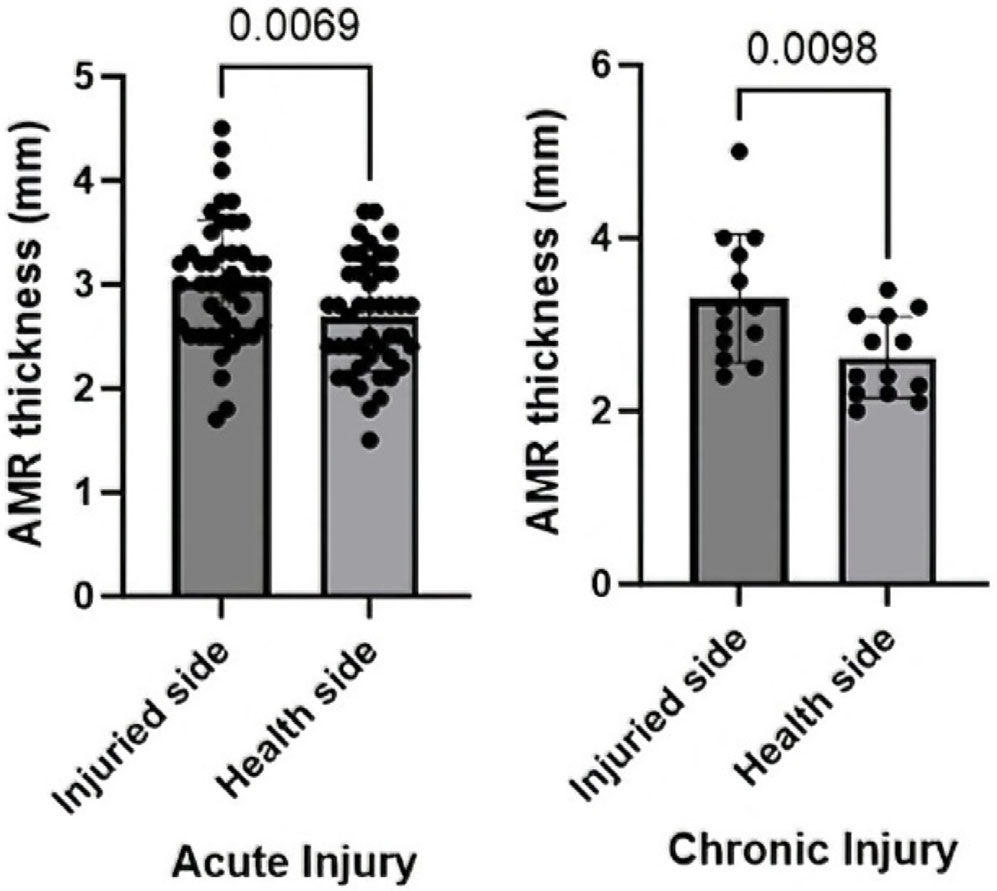
Affected and unaffected AMR thickness in acute injury (<6 weeks) and chronic injury (>6 weeks). (AMR, anteromedial retinaculum.)

The data analysis of body mass index, CTS, and MTS showed no significant differences among the 3 groups. MFCW, NW, NWI, LFCR, MTD, and LTS showed significant differences among the 3 groups (*P* < .05) (Table 3).

**TABLE 3:** Comparison of Demographics and Bone Morphology Factors Among the AMRI, NAMRI, and Control Groups

**Group**	**NAMRI (n = 25)**	**AMRI (n = 58)**	**Control (n = 58)**	***P*** **Value**
Sex (male/female)	15/10	37/21	37/21	‐
BMI	26.06 ± 2.90	25.47 ± 3.61	25.87 ± 4.28	.762
LFCR	0.668 ± 0.035	0.672 ± 0.039	0.644 ± 0.043	**.006**
CTS	3.4 ± 8.2	2.2 ± 8.1	4.9 ± 5.9	.140
NW	1.73 ± 0.24	1.79 ± 0.29	2.01 ± 0.37	**<.001**
MFCW	7.03 ± 0.37	6.72 ± 0.55	7.35 ± 0.51	**<.001**
NWI	0.248 ± 0.035	0.265 ± 0.036	0.273 ± 0.044	**.024**
MTD	0.36 ± 0.08	0.36 ± 0.07	0.31 ± 0.06	**<.001**
LTS	8.1 ± 2.3	8.5 ± 2.6	6.4 ± 2.9	**.002**
MTS	7.4 ± 2.5	7.4 ± 2.6	6.7 ± 2.8	.251

*Note:* The bold numerical values mean *P* < .05.

AMRI, anteromedial rotatory instability; BMI, body mass index; CTS, coronal tibial slope; LFCR, lateral femoral condyle ratio; LTS, lateral tibial posterior slope; MFCW, medial femoral condyle width; MTD, medial tibial depth; MTS, medial tibial posterior slope; NAMRI, nonanteromedial rotatory instability; NW, notch width; NWI, notch width index.

The AMRI group had significantly smaller MFCW and NW than the control group (*P* < .05) (Figure 6). The AMRI group had significantly larger LFCR, LTS, and MTD than the control group (*P* < .05). The NAMRI group had significantly larger LFCR, LTS, and MTD than the control group (*P* < .05). The NAMRI group had smaller NW, MFCW, and NWI than the control group. Between the AMRI and NAMRI groups, the MFCW was significantly smaller in the AMRI group (AMRI: 6.72 ± 0.55 vs NAMRI: 7.03 ± 0.37, *P* = .0332). CTS and MTS showed no significant difference in any pairwise comparison among the 3 groups.

**FIGURE 6:**
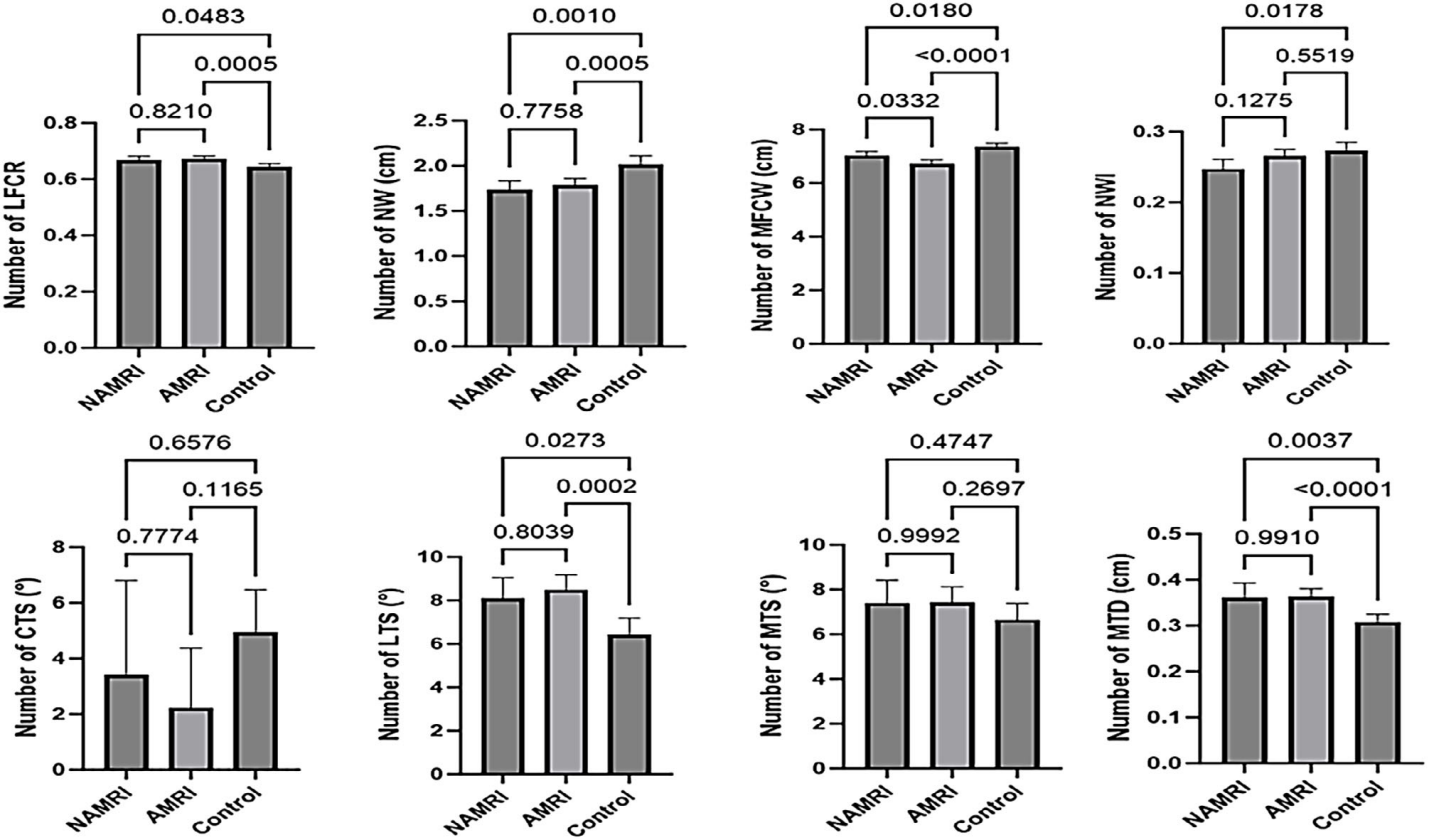
Histogram of the between‐group differences in bone morphological factors among the NAMRI, AMRI, and control groups. (AMRI, anteromedial rotatory instability; CTS, coronal tibial slope; LFCR, lateral femoral condyle ratio; LTS, lateral tibial posterior slope; MFCW, medial femoral condyle width; MTD, medial tibial depth; MTS, medial tibial posterior slope; NAMRI, nonanteromedial rotatory instability; NW, notch width; NWI, notch width index.)

ROC curve analysis for MFCW (Figure 7) between the AMRI group and the control group showed an area under the ROC curve of 0.791 with an optimal cutoff value of 7.5 mm. Between the NAMRI and AMRI groups, the area under the ROC curve for MFCW was 0.655 with an optimal cutoff of 6.5 mm (Table 4).

**FIGURE 7:**
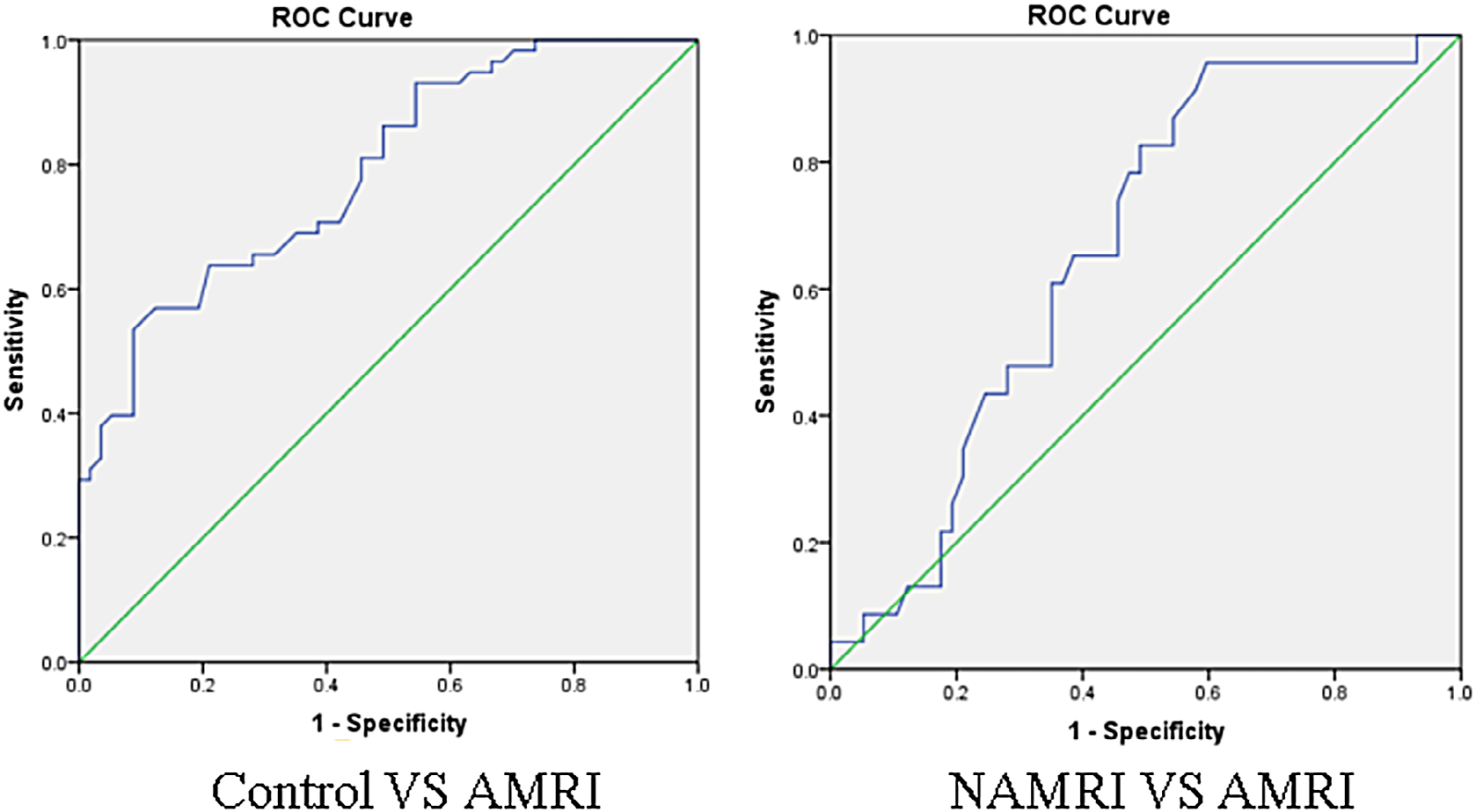
ROC curve of the MFCW. (AMRI, anteromedial rotatory instability; MFCW, medial femoral condyle width; NAMRI, nonanteromedial rotatory instability; ROC, receiver operating characteristic.)

**TABLE 4:** Cutoff Values and Their AUC for Identifying AMRI

**Group**	**Cutoff**	**Sensitivity**	**Specificity**	**AUC**
Control vs AMRI	7.35	0.552	0.897	0.791
NAMRI vs AMRI	6.5	0.960	0.414	0.655

AMRI, anteromedial rotatory instability; AUC, area under the receiver operating characteristic curve; NAMRI, nonanteromedial rotatory instability.

## DISCUSSION

The most important finding of this study is that the patients with AMRI have a smaller MFCW compared with the NAMRI group and the control group. Currently, there is limited research directly investigating risk factors in AMRI patients. This study used the anterior drawer test and Slocum test to diagnose the AMRI condition, grouping patients based on physical examination result to investigate differences in bone morphology on preoperative MRI and underlying injuries to the medial knee structures.

Previous studies have suggested that a larger LFCR, indicating a more prominent posterolateral femoral condyle, may prolong the relatively flat central area of the lateral femoral condyle. This could lead to greater femoral displacement during relative motion between the femur and tibia.^25^ A narrower NW may increase the risk of ACL impingement during movement, thereby elevating the likelihood of ACL injury.^26^ Additionally, greater LTS and MTS are associated with increased anterior tibial translation,^14^, ^27^ potentially increasing axial displacement and injury risk during activity. These bone morphological factors have been identified as risk factors for ACL injury. However, their relation with AMRI has not been previously established. The results of LTS and MTS on MRI as proposed by Hudek et al. tend to smaller values compared with results on radiographs.^28^ This may account for the lack of statistically significant differences in LTS and MTS observed across all 3 groups in the analysis. Our data indicated that compared with control patients, AMRI patients have a larger LFCR (more posteriorly prominent lateral femoral condyle), smaller MFCW and NW, and larger LTS and MTD. Notably, significant pairwise differences in MFCW were observed among the 3 patient groups, with the AMRI group showing significantly smaller MFCW than both the control and NAMRI groups. This suggests that reduced MFCW is a characteristic bone morphological feature in AMRI patients. A 3‐dimensional finite element study suggested that smaller femoral condyles might reduce the moment arm of ligamentous structures (including ACL, MCL, posterior oblique ligament, and anterolateral ligament) during internal and external rotation in women.^25^ This could increase stress on these ligaments and potentially make individuals with smaller femoral condyles more susceptible to injury during knee rotation.

Most current biomechanical and anatomical studies indicated that the ACL and MCL play crucial roles in controlling AMRI.^29^, ^30^, ^31^ The ACL and sMCL, respectively, provide 63% to 78% and 7% to 4% of the restraint against anterior tibial translation during knee flexion from 0° to 90°. At 90° of flexion, the dMCL provides the primary restraint against external tibial rotation.^31^ However, our statistical analysis of AMR thickness showed that compared with the healthy contralateral knee, the injured side AMR was thicker in both the chronic injury group and the acute injury group. This finding implies that AMRI may also involve AMR structural injury.

The traditional understanding of AMRI largely attributes the primary control to the sMCL and posteromedial complex, in addition to the ACL injury.^5^ However, our preoperative ultrasound examination of AMRI patients revealed that the most common combined injuries were the dMCL (86.2%) and AMR (63.5%), in addition to ACL injury. Only a minority exhibited sMCL injury (31%) in our study. When there is inconsistency between the preoperative ultrasound and preoperative MRI findings regarding injuries to the sMCL and dMCL, the preoperative MRI results are considered definitive. Studies have shown that the sensitivity and specificity of MRI for detecting grade II or III MCL injuries are 68% and 90%.^32^ Grunenberg et al. precisely identified the location of the AMR on MRI.^9^ Because of the inability to accurately identify a clear independent structure on MRI like Grunenberg et al. this study did not count the damage to the patient's AMR based on MRI. This finding suggested that sMCL injury condition does not fully correlate with AMRI condition. Kennedy and Fowler proposed a possible mechanism for AMRI that the dMCL is the first structure torn during external rotation and valgus force.^33^ Increased external tibial rotation because of dMCL injury places additional stress on the sMCL and ACL, positioning the dMCL as the primary lesion in AMRI. A recent video analysis study of injury mechanisms indicated that nearly 60% of ACL injuries in soccer players occurred under external rotation conditions.^34^ In addition to MCL injury, this mechanism implied that anteromedial injuries might be more common than posteromedial injuries.^31^ Another recent study highlighted the importance of additional anteromedial reconstruction for knees with excessive AMRI.^7^ Biomechanical studies have shown that only additional anteromedial reconstruction can resist excessive AMRI.^22^ Therefore, AMRI should primarily be determined by the injury condition of the ACL, dMCL, and AMR. This study only observed the injured structures in AMRI patients but did not explore the causal relation or biomechanical mechanisms linking AMRI to these structural injuries.

A strength of our study is the inclusion of a group with isolated ACL injury and a negative Slocum test (NAMRI group). Although the sample size of this group is relatively small, comparing 3 groups enables a clearer understanding of the bone morphological differences among patients with no ligament injury, isolated ACL injury, and AMRI. Another advantage is the application of ultrasound to assess injured structures in patients with AMRI condition and the identification of a distinct ligament‐like structure (AMR), via ultrasound.

### Limitations

This study has a number of limitations that must be discussed. The sample size of this study is relatively small. However, the inclusion of 3 groups for comparison allows for meaningful contrasts in bone morphological factors among patients with no ligament injury, isolated ACL injury, and AMRI. Because of the small sample size, we were unable to desegregate the data by sex as recommended by the sex and gender‐based analyses guidelines. This is a single‐center study involving only Asian patients, and the findings may not be generalizable to other ethnicities. Among the AMRI patients selected for this study, 22.4% underwent MRI examinations more than 3 weeks after the time of injury. These delayed MRI scans may introduce bias into the results.

## CONCLUSIONS

A smaller MFCW is associated with AMRI in patients with dMCL and AMR injuries compared with NAMRI patients. The injury pattern in AMRI is predominantly characterized by combined lesions of the ACL and dMCL + AMR.

## DISCLOSURES

The author (J.D.) declares the following financial interests/personal relationships which may be considered as potential competing interests: J.D. reports financial support provided by the Foundation for Innovative Research Groups of the National Natural Science Foundation of China. The other authors (S.W., R.Z., Y.L., Y.Z., Z.L.) declare that they have no known competing financial interests or personal relationships that could have appeared to influence the work reported in this article.

## FUNDING

This study was supported by the “14th Five‐Year” clinical medicine Innovation Research Team Support Program of Hebei Medical University (2022LCTD‐B25), the National Natural Science Foundation of China (No. 82471596), and the National Natural Science Foundation of China (No. 82172460).
